# Hollow Salt Prepared Through Spray Drying with Alginate Enhances Salinity Perception to Reduce Sodium Intake

**DOI:** 10.3390/foods14010019

**Published:** 2024-12-25

**Authors:** Qian Jiang, Jiayi Yan, Chen Song, Yunning Yang, Guangyuan Chen, Fanhua Kong, Jingfeng Yang, Shuang Song

**Affiliations:** SKL of Marine Food Processing & Safety Control, National Engineering Research Center of Seafood, Liaoning Key Laboratory of Food Nutrition and Health, School of Food Science and Technology, Dalian Polytechnic University, Dalian 116034, China; rosyqqxi@163.com (Q.J.); 13934546952@163.com (J.Y.); sczoe86@163.com (C.S.); yangyn012@163.com (Y.Y.); noahchen2022@163.com (G.C.); kkong0930@163.com (F.K.); yjfgo@163.com (J.Y.)

**Keywords:** alginate, saltiness, spray drying, hollow salt, salt reduction

## Abstract

Currently, high-salt diets have become one of the world’s biggest dietary crisis and long-term high-salt diets are seriously detrimental to human health. In response to this situation, the present study proposed a saltiness enhancement strategy using alginate, which is a dietary fibre from brown algae and has many health benefits, such as regulating intestinal microbiota, anti-hypertension and anti-obesity. The comparison of alginates with different viscosities showed that alginate of 1000–1500 cps at a concentration of 1.25 g/L could enhance the saltiness of NaCl solution by 11.5%. Then, a solid salt was prepared through spray drying with 4.83% of this alginate, and its structure was characterised by X-Ray diffraction, Fourier transform infrared spectroscopy, scanning electron microscopy and energy dispersive spectroscopy to confirm its hollow structure with a particle size of 6.25 ± 2.26 μm as well as its crystal structure similar to original NaCl. Moreover, the conductivity monition revealed that the hollow salt exhibited a more rapid dissolution in water and its alginate component increased the adhesive retention of sodium ions on the tongue surface, which both effectively enhanced the sensory perception. Finally, as revealed by the sensory evaluation, the prepared hollow salt showed higher saltiness than that of original table salt and it could reduce sodium intake by 29%. Thus, the hollow salt prepared with alginate in the present study has potential for salt reduction.

## 1. Introduction

Studies have shown that people’s average daily salt intake far exceeds the amount needed to meet basic physiological functions [1]. Globally, the average daily sodium intake is between 3.54 g and 4.72 g, far exceeding the maximum of 2 g/day recommended by the World Health Organization (WHO) [2]. However, excessive sodium intake can cause an elevation of blood pressure, which further leads to cardiovascular diseases, such as hypertension and coronary heart disease [3,4]. In addition, a high-sodium diet could alter the composition of the human gut microbiota, affect cerebrovascular morphology and structure and increase the risk of Alzheimer’s disease and osteoporosis [5,6,7]. In response to this situation, the WHO has set the goal of reducing salt intake by 30 percent by 2025 [8].

Reducing excessive salt intake without hindering the saltiness perception has received a great deal of attention in recent years. The food industry and researchers have been exploring ways to reduce sodium intake while maintaining the nutritional value and flavour of foods, and some chemical and physical salt reduction strategies have been applied. Chemical salt reduction strategies include the salt substitution, the application of saltiness potentiators and the modulation of sodium absorption in the body. Salt substitutes refer to substances with less sodium content possessing a similar salty taste to NaCl, so it can replace NaCl to some degree. Currently, commonly used salt substituents include potassium chloride, magnesium chloride, magnesium sulphate, potassium lactate, calcium lactate and so on. Saltiness potentiators are a kind of substance without inherent salty taste, such as glycine, glycine monoethyl ester, lactic acid, 1-ornithine and glutamic acid, etc., and they can make salt taste receptors more sensitive so as to improve the saltiness perception [9]. In addition, reducing the sodium absorption is also an important method. Some polysaccharides, such as carboxymethyl cellulose (CMC) and gum arabic (GA), may interact with sodium ions, thereby potentially promoting sodium excretion [2]. It has been shown that the addition of chitosan and cellulose to sausages could promote sodium excretion in urine and faeces, thereby reducing sodium absorption in mice [10]. In addition to the above strategies, the structure adjustment of solid salt including changes in the salt particle size and shape and fabrication of hollow structures is also an effective method for salt reduction through increasing the surface area and accelerating the dissolution rate of solid salt [11,12,13]. By controlling the evaporation process and mixing with other macromolecule substances, salt crystals with hollow structures can be obtained, maximising their surface area to achieve a saltier taste [2]. The procedure to fabricate hollow salt includes mixing the salt microcrystals after grinding, drying and sieving with the prepared hydrogel beads, and then drying at low temperature to obtain hollow salt microcapsules [14]. However, spray drying is the preferred method for the preparation of hollow salts in the food industry due to its low cost and high efficiency.

Some polysaccharides such as CMC, hyaluronic acid, GA, λ-carrageenan and starch have been shown to enhance saltiness perception by prolonging the retention time of sodium in the oral cavity [10,15,16,17,18]. Alginate is a natural polysaccharide consisting of β-D-mannuronic acid (M) and α-L-guluronic acid (G). Due to its unique molecular structure and hydrophilicity, alginate is often added to food products as thickeners and stabilisers. It has the advantages of low cost, easy availability and non-toxic side effects, and is widely used in the food industry [19]. In addition, alginate has a variety of functional activities such as antioxidant, antibacterial, antiviral and immunomodulatory effects [20]. As a dietary fibre from brown algae, alginate also shows anti-obesity efficacy and could regulate intestinal microbiota [21,22]. Moreover, it has antihypertensive effects [20], suggesting its potential for elevated blood pressure associated with excessive sodium intake. Thus, in the present study, alginate was chosen for the fabrication of hollow salt.

The present study aimed to prepare a hollow salt with alginate for sodium reduction. Firstly, alginate with different viscosities at different concentrations were compared by testing their saltiness enhancement effects through electronic tongue determination and sensory evaluation. Then, a hollow salt was prepared with alginate through spray drying, and its structure was characterised by X-Ray diffraction, Fourier transform infrared spectroscopy, scanning electron microscopy and energy dispersive spectroscopy. Meanwhile, the underlying mechanism of the saltiness enhancement effect of alginate was explored by analysing the dissolution efficiency of the prepared solid salt and the adhesive retention of sodium ions on the surface of the tongue. Finally, the sodium reduction effect of the prepared hollow salt was evaluated by the sensory evaluation.

## 2. Materials and Methods

### 2.1. Materials

Three alginates with a high viscosity of 1000–1500 centipoise per second (cps), medium viscosity of 200–600 cps and low viscosity of 20–100 cps were provided by Qingdao Mingyue Seaweed Group Co., Ltd. (Qingdao, China). The table salt, NaCl, was brought from Guangdong Hongyuan Food Ingredients Shop (Shantou, China). Fresh pig tongues were obtained from a local market (Dalian, China). Artificial saliva was obtained from Beijing Reagan Biotechnology Co., Ltd. (Beijing, China). Fluorescein sodium was brought from Shanghai Macklin Biochemical Technology Co., Ltd. (Shanghai, China). Alginate and NaCl were of food grade and other reagents were of analytical grade.

### 2.2. Sensory Evaluation of Salt Solutions

Twenty volunteers without taste deficiencies aged 20–30 were recruited. Before conducting the formal sensory evaluation experiment, a selection process was required, and it included a basic taste experiment, a saltiness threshold experiment, a differentiation experiment, and a sorting and grading experiment [23]. In the basic taste experiment, evaluators were required to match the correct tastes of six samples containing basic tastes, and individuals with an accuracy rate higher than 75% were selected for the next experiment. In the saltiness threshold experiment, evaluators tasted the NaCl solution in the order of increasing concentration, and individuals with a detection threshold less than 1 g/L were selected to participate in subsequent experiments. In the differentiation experiment, evaluators were required to select the different sample from three samples. In the sorting and grading experiment, evaluators were required to sort the samples in increasing order of saltiness, and individuals finishing the task with no or only one mistake were selected for subsequent experiments. After a series of the above experiments, 8 qualified sensory evaluators were selected for the subsequent sensory evaluation. The selected sensory evaluators started to conduct the determination of the standard concentration of NaCl salty taste, and the sample concentration of NaCl standard salty taste could be obtained through graded sensory experiments. Then, the sensory evaluators were trained through the standard samples, so that the evaluators could be familiar with and distinguish the differences between the different taste intensities.

A 5 g/L solution of NaCl was used as the control. Firstly, a series of alginate solutions were prepared by dissolving different viscosities of alginate in water, with concentrations of 0.25 g/L, 0.75 g/L and 1.25 g/L for each viscosity of alginate solution. The solutions were then heated and stirred at 80 °C to ensure complete dissolution. After that, different amounts of NaCl were added to them to keep the sodium content in the solutions consistent with that of the control, and the final mass ratios of alginate and NaCl were 1:19.7, 1:6.37 and 1:3.70. The mixed solutions were left at room temperature overnight. Sensory evaluation was carried out for each mixed solution and the control. Prior to sensory evaluation, the samples were poured into tasting cups which were then randomly distributed to the evaluators. NaCl solution of 3 g/L was used as the standard reference (5 points) and the intensity of the salty taste of each sample was scored within a range between 1 point and 9 points.

In addition, all sensory experiments were conducted in a sensory evaluation room where the temperature was maintained at 20–24 °C and the relative humidity was maintained at 45–65% to ensure the comfort of the evaluators and the accuracy of the taste. The light in the room was sufficient, bright and uniform, and the air in the room was fresh and free from odours.

### 2.3. Electronic Tongue Determination

The saltiness value of each solution was determined using a TS-5000Z electronic tongue (Beijing Ying Sheng Hengtai Science and Technology Co., Ltd., Beijing, China) containing five taste sensors and two reference electrodes. Data collection started after the sensors were immersed in the samples and the measurements were repeated 5 times for each sample.

### 2.4. Preparation of Solid Salts

The mixture of high viscosity alginate and NaCl at a ratio of 1:19.7 was prepared. After the mixture was thoroughly mixed, it was sprayed dry using a spray dryer (ADL311, Chongqing Yamato Technology Co., Ltd., Chongqing, China). The inlet temperature of the spray dryer was set at 160 °C and the outlet temperature was set at 60 °C. In addition, the atomization pressure was maintained at 0.15 MPa and the feeding rate was 10 mL/min. After spray drying, the solid powder was collected as alginate-NaCl sample.

### 2.5. Characterisation of Solid Salts

The crystal structure of alginate–NaCl as well as spray-dried NaCl was determined using an X-Ray diffractometer (XRD-7000S, Shimadzu, Kyoto, Japan). The X-Ray diffraction patterns were examined with minor modifications following the previously reported methods [24]. The XRD tests were performed with the voltage set at 40 kV and the current set at 30 mA and scanned at a 2θ angle between 10° and 90°.

For the Fourier transform infrared (FTIR) spectroscopy analysis, alginate, NaCl and alginate-NaCl (2 mg) were mixed and ground with KBr (100 mg) individually. The FTIR spectra were recorded at room temperature using a FTIR spectrometer (Spectrum two, Platinum Elmer Instruments Ltd., Shanghai, China) in the range of 4000–400 cm^−1^.

Scanning electron microscopy (SEM) and energy dispersive spectroscopy (EDS) were performed with reference to previously reported experimental methods [25,26]. A JSM-7800F thermal field emission scanning electron microscope system (Nihon Electronics, Tokyo, Japan) equipped with an X-Max50 energy spectroscopy (Oxford Instruments, Oxford, UK) and a backscattered electron diffraction analyser (Nihon Electronics, Tokyo, Japan) was used to observe the microstructures of alginate–NaCl, spray-dried NaCl, and the original NaCl at different magnifications. The SEM was used in backscattered electron mode, and each sample was fixed on a sample stage and sprayed with gold for 1 min under vacuum to form a conductive surface. Furthermore, Image J software (version 1.53 q) was used to determine the particle size of alginate–NaCl. Then, SEM was combined with energy spectroscopy to analyse the content and distribution of the elements on the surface of alginate–NaCl.

### 2.6. Determination of Sodium Retention on the Tongue In Vitro

Two experiments were performed to determine the adhesion retention of sodium on pig tongues in vitro with reference to the reported experimental method [27] with slight modifications. Pigs were slaughtered within 24 h and their tongues were kept on ice during transport. Most of the muscle and tissue was removed from the lower side of the pig tongue, and the epithelial layer was placed in self-sealing bags and stored at −20 °C. When needed, the tissues were thawed at room temperature and cut into sections of approximately 1 cm^2^ (approximately 0.7–0.8 mm thick), so that these slices could adhere to the microscope slides and at the same time allow better transmission of light from the microscope.

In the first experiment, 0.3 wt% high-viscosity alginate (HV-alginate) solution was prepared and mixed with 0.01 wt% fluorescein sodium solution. The control group was a fluorescein sodium solution without alginate. A 20 μL drop of the solution was placed on the surface of the pig tongue and left to stand for 30 s. The tongue was then placed at an angle of 45° and rinsed with different volumes of artificial saliva, controlling the flow rate to 6 mL/min. Photographs of fluorescently stained pig tongues were taken using a Nikon Ti-s fluorescence inverted microscope (Nikon, Tokyo, Japan) and then analysed using Image J software to quantify the fluorescence intensity. The experiment was repeated three times for each sample.

In the second experiment, a 20 μL volume of the mixture of alginate and NaCl at a ratio of 1:19.7 or the NaCl solution, whose sodium contents were both 6.825 g/L, was dropped on the surface of the pig tongues, and the next experimental procedure was the same as above. Then, the tongues were rinsed with 10 mL and 20 mL of artificial saliva. The sodium content in the rinsed artificial saliva was measured at 589.0 nm using a ZA3000 atomic absorption spectrophotometer (Hitachi, Tokyo, Japan).

### 2.7. Dissolution Kinetics of Salt Particles in Water

Salt dissolution was evaluated by measuring the conductivity of the solution of salt particles within 30 s, referring to the reported experimental method [28] with slight modifications. Briefly, 0.2 g of alginate–NaCl and NaCl were dispersed into 50 mL of water at 20 °C with stirring at 200 rpm. The conductivity values were recorded every 5 s using a conductivity electrode (Shanghai Youke Instrumentation Co., Ltd., Shanghai, China).

### 2.8. Sensory Evaluation of Solid Salts

The sensory evaluation was carried out with reference to the reported experimental method [25] with minor modifications. Briefly, 15 mg of the prepared alginate–NaCl or different amounts of NaCl were sprinkled on 1 g instant noodles. Then, the prepared samples were numbered with three random digits and distributed to the sensory evaluators in a random order. Prior to tasting the samples and between each tasting, the evaluators were required to rinse their mouths with purified water. The saltiness of the sample containing 1.5% NaCl was set to 5 scores, and each solid salt was scored according to the intensity of salty taste on a scale of 1–10.

### 2.9. Statistical Analysis

All experiments were repeated at least three times, and all results were expressed as mean ± SD. Data were analysed using SPSS version 19.0 software and compared using one-way ANOVA. Before performing one-way ANOVA, normality tests (Shapiro–Wilk) were performed. Values of *p* < 0.05 were considered statistically significant.

## 3. Results and Discussions

### 3.1. Enhancement of Solution Saltiness by Alginates

**Figure 1:**
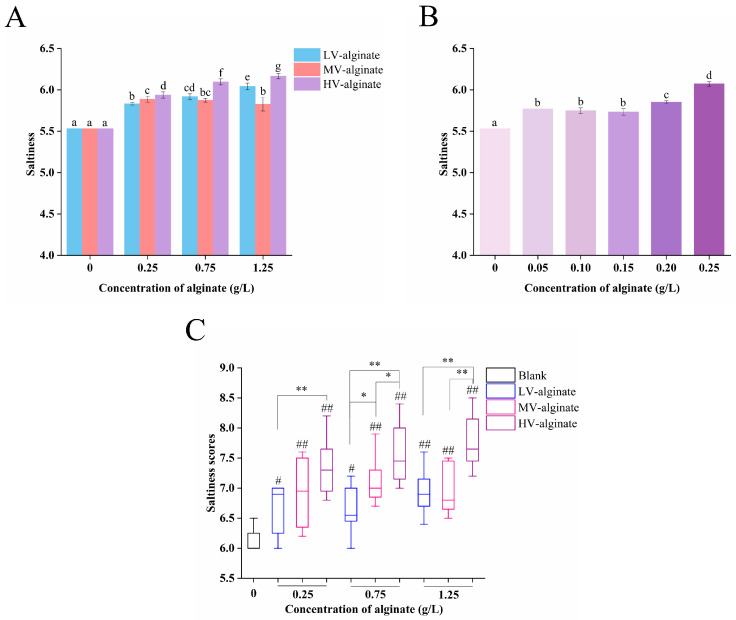
Saltiness values of NaCl solutions containing alginates of different viscosities obtained by electronic tongue measurement, where different letters indicate significant differences, *p* < 0.05 (**A**). Saltiness values of solutions containing HV-alginate at different concentrations, and different letters indicate significant differences, *p* < 0.05 (**B**). Saltiness scores of mixed solutions containing alginates of different viscosities at different concentrations obtained by sensory evaluation, where # indicates the significant difference between each mixed solution and the control (0 g/L alginate), and * indicates the significant difference between the mixed solutions containing alginate with different viscosities at the same concentration. # *p* < 0.05, ## *p* < 0.01, * *p* < 0.05, ** *p* < 0.01 (**C**).

The effects of alginates with different viscosities on the saltiness of NaCl solutions were compared by sensory evaluation and electronic tongue analysis. As shown in Figure 1A, the results of electronic tongue measurement showed that the NaCl solutions containing alginates with different viscosities at concentrations from 0.25 g/L to 1.25 g/L all significantly increased the saltiness compared with the control group without alginate. The saltiness of the NaCl solutions increased with the increase in alginate viscosity at alginate concentration of 0.25 g/L but this trend did not occur at alginate concentrations of 0.75 g/L and 1.25 g/L. However, it could be included that among the three alginate samples, HV-alginate showed the strongest saltiness enhancing effect. The saltiness enhancement by HV-alginate at low concentrations was further investigated using electronic tongue. As shown in Figure 1B, the saltiness of the solution was significantly increased when the concentration of HV-alginate was 0.05 g/L compared to the blank control. However, there was no significant difference in the saltiness among solutions with 0.05 g/L, 0.10 g/L and 0.15 g/L HV-alginate. When the concentration of HV-alginate increased to 0.20 g/L, the saltiness raised significantly. Furthermore, the saltiness of the solution containing 0.25 g/L HV-alginate was even higher. As shown in Figure 1C and Table S1, the results of the sensory evaluation were similar to those of the electronic tongue measurement, and they all demonstrated that alginates of different viscosities could increase the saltiness of NaCl solution. Notably, the saltiness-enhancing effect of alginate was positively related to its viscosity. The saltiness of the solutions with HV-alginate was stronger compared to that of low-viscosity alginate (LV-alginate) at all the detected concentrations, and the saltiness-enhancing effect of HV-alginate was better than that of medium-viscosity alginate (MV-alginate) at 0.75 g/L and 1.25 g/L.

It has been reported that some polysaccharides can enhance the saltiness of liquid or semi-solid foods, such as CMC, GA and carrageenan. For example, it has been found that low-viscosity (5–12 cps) CMC could enhance the saltiness of NaCl solution [29], and CMC with a molecular weight of 140 kDa and a concentration of 2.6% (*w*/*w*) could adjust the saltiness of NaCl solution and the saltiness perception in the oral cavity could be enhanced due to the adhesion properties [27]. In addition, protease-hydrolysed GA at a concentration of 0.3 wt% effectively enhanced the saltiness perception [17]. Moreover, in a low-sodium salt system composed of NaCl and KCl, the addition of carrageenan (Mw = 367.6 kDa) not only reduced the bitter taste of KCl but also enhanced the salty taste of the system, which could be attributed to the capability of carrageenan to prolong the residence of sodium ion on the surface of the tongue, thus enhancing the saltiness perception. Meanwhile, comparing the effects of carrageenans with different molecular weights (9 KDa-367.6 KDa) on the saltiness of the solution, it was found that those with molecular weights higher than 51.6 kDa could enhance the saltiness of the salt solutions significantly [30]. Thus, the above results indicate that viscosity was an important factor for polysaccharides to enhance the saltiness perception.

### 3.2. X-Ray Diffraction Analysis of Solid Salts

**Figure 2:**
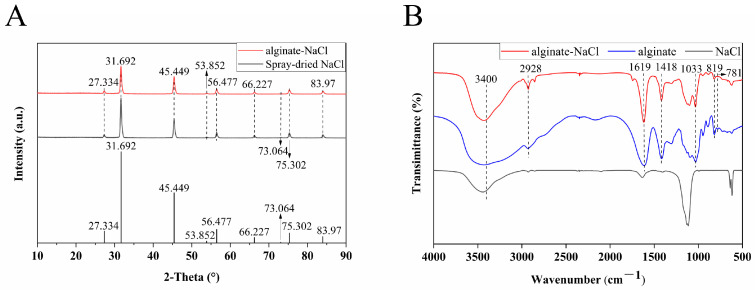
X-Ray diffraction patterns (**A**) and FTIR spectra (**B**) for structural analysis of solid salts.

Based on the above results, HV-alginate at a concentration of 0.25 g/L was selected for the solid salt fabrication through spray drying, and the prepared solid salt (alginate-NaCl) was white powder containing 4.83% HV-alginate. X-Ray diffraction was used to determine the crystal structure of alginate–NaCl as well as spray-dried NaCl, and the obtained diffraction peaks were analysed and compared with the standard card of NaCl. As shown in Figure 2A, XRD results showed that alginate–NaCl contained mainly NaCl crystals which is similar to that of spray-dried NaCl. This indicated that the addition of HV-alginate and the spray drying process did not destroy the crystal structure of NaCl. Chindapan et al. prepared a solid salt mixture of NaCl and KCl using the spray drying technique and found that neither spray drying nor the interaction between KCl and NaCl had any effect on the crystal structure of the mixture of KCl and NaCl [31], which is similar to the results of the present study. It has been reported that the crystal structure is related to the inherent dissolution rate of the crystal [32]. In the present study, no alteration occurred in crystal structure of alginate–NaCl as compared to NaCl, so its dissolution property was not affected by crystal structure theoretically.

### 3.3. Fourier Transform Infrared Spectroscopy Analysis

The FTIR spectrum of alginate–NaCl was shown in Figure 2B and compared with those of alginate and NaCl. In the infrared spectrum of alginate–NaCl, the characteristic peaks at 1619 cm^−1^ and 1418 cm^−1^ correspond to the asymmetric and symmetric stretching vibrations of the carboxyl C=O, respectively. The small peak at 2928 cm^−1^ and the peak at 1033 cm^−1^ correspond to the C-H and C-O stretching vibrations, respectively. Moreover, the peaks at 781 cm^−1^ and 819 cm^−1^ are characteristic peaks of the G and M groups, respectively [33,34]. Finally, the broad absorption peak at around 3400 cm^−1^ could be attributed to the lattice vibration in the NaCl structure and the O-H stretching vibration in the alginate structure [33,34]. The specific results were shown in Table 1. Notably, these absorption bands are all the summation of those of alginate and NaCl, and no obvious peak shift caused by their mix, indicating no change in the chemical bonds or NaCl lattice.

### 3.4. SEM Analysis of Solid Salts

**Figure 3:**
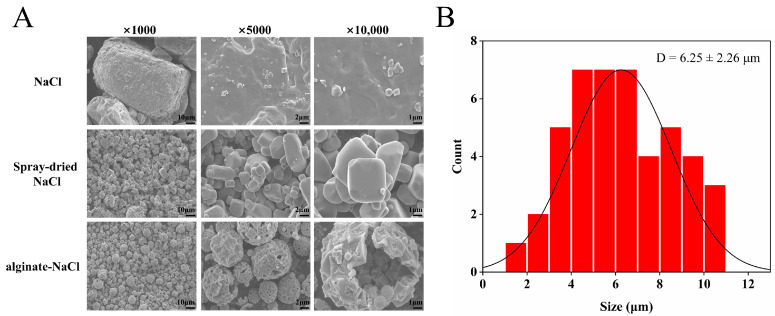
Scanning electron micrographs of different salt particles (**A**) and particle size distribution of alginate–NaCl (**B**).

The microstructure of the solid salt was observed via SEM at different magnifications. As shown in Figure 3A, the original NaCl presented a solid crystalline cubic morphology, and after spray drying, it formed smaller crystalline particles with a higher specific surface area, but commonly aggregated. In contrast, the solid salt containing HV-alginate showed a hollow spherical structure without coalescence between particles and its particle size was approximately 6.25 ± 2.26 μm (*n* = 45), as shown in Figure 3B.

The hollow structure of NaCl can be prepared by spray drying after adding potassium chloride, chitosan, saponin or other substances to NaCl solution [25,26,31]. For example, hollow salt microspheres with an average particle size of 10–20 μm were prepared by mixing maltodextrin with NaCl [35] and hollow salt microspheres produced by spray drying technique using quillaja saponin nano-emulsion droplets and table salt as raw materials were basically below 16 μm in size [25]. In addition, alginate–NaCl obtained in the present study possessed a relatively small size, and it has been reported that the small size of hollow salt is beneficial for its enhancement on saltiness [2].

### 3.5. EDS Analysis of Solid Salts

**Figure 4:**
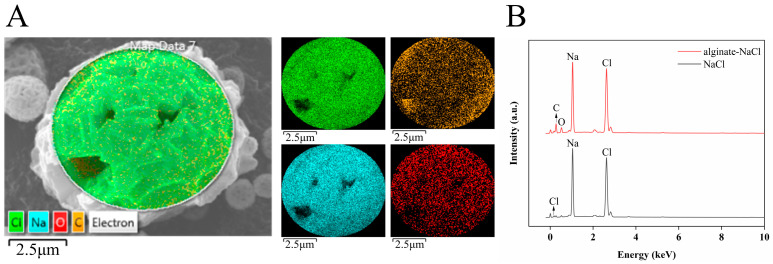
Distribution of elements on the surface of alginate–NaCl (**A**) and elemental signals on the surfaces of alginate–NaCl and NaCl (**B**).

In order to investigate the distribution of alginate in alginate–NaCl, EDS was used to analyse the surface elemental distribution of alginate–NaCl. As shown in Figure 4A, four elements, Na, Cl, C and O, exhibited on the hollow salt particle. Among them, C and O were from alginate and could reflect the distribution of alginate. It can be found that alginate is evenly distributed on an alginate–NaCl particle. Moreover, as shown in Figure 4B, Na and Cl were the major components in alginate–NaCl while only a trace of C and O existed, which is consistent with the minor addition amount of alginate (4.83%). In the previous study, the air-drying method was employed to prepare low-sodium salts using a mixed system of carrageenan, NaCl and KCl, but an uneven distribution of Na and K was found on their surfaces [30]. It is initially speculated that the spray drying process may have advantages in the uniform distribution of elements compared to the air-drying method.

### 3.6. Sodium Retention In Vitro

**Figure 5:**
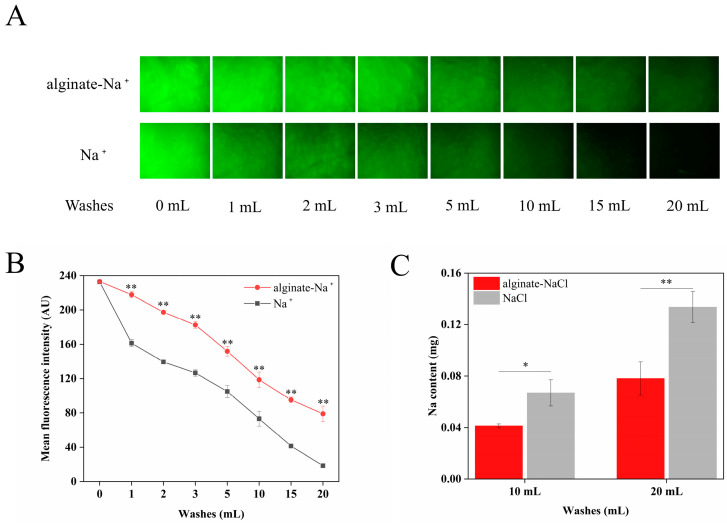
Fluorescence pictures of porcine tongues in vitro (**A**). Fluorescence intensity obtained using Image J, where * indicates significant differences in fluorescence intensity at the same volume of artificial saliva, ** *p* < 0.01 (**B**). The sodium content of the rinsed artificial saliva, where * indicates significant differences, * *p* < 0.05, ** *p* < 0.01 (**C**).

In order to reveal the action mechanism of alginate to enhance the salty taste of the solution, a sodium retention experiment was carried out using porcine tongues. As shown in Figure 5A,B, the fluorescence intensity of the alginate and control groups gradually diminished as artificial saliva rinsed. Notably, the fluorescence intensity of the alginate group tended to diminish more slowly compared to that of the control group. After rinsing with 20 mL of artificial saliva, there was little fluorescein sodium retained on the porcine tongues in the control group while more fluorescein sodium was observed in the alginate group. This indicates that alginate effectively prevented the loss of fluorescein sodium and enhanced the adhesive retention of substances on the surface of the tongues.

Moreover, after rinsing the porcine tongue with 10 mL and 20 mL artificial saliva, the rinsed sodium contents were determined and shown in Figure 5C. More artificial saliva could rinse more sodium. Notably, however, the released sodium in the alginate–NaCl group was lower than that in the NaCl group no matter how much artificial saliva was applied. This further confirms that alginate enhanced the adhesive retention of sodium ions on the surface of the tongues.

It has been reported that the adhesive behaviour of some polysaccharides, such as CMC, enhances the retention of sodium ions on the tongue, which is an important factor influencing the saltiness perception in the oral cavity [15,27]. The substantial amount of carboxyl groups in alginate structure can contribute to mucoadhesion through hydrogen bonds and van der Waals forces with the oligosaccharide side chains of mucin, which is similar to the that of CMC [27].

### 3.7. Dissolution Kinetics of Salt Particles in Water

**Figure 6:**
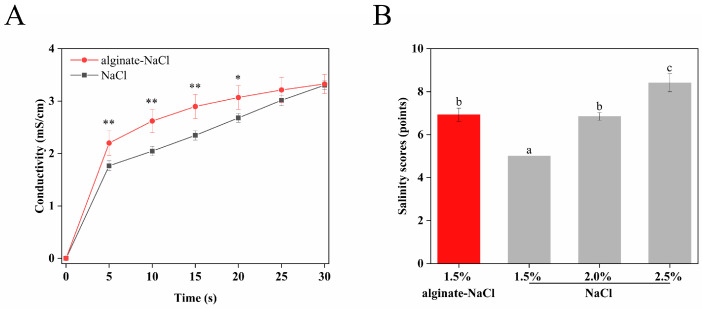
Dissolution curves of alginate–NaCl and NaCl in water over time, where * indicates a significant difference in conductivity between alginate–NaCl and NaCl at the same time, * *p* < 0.05, ** *p* < 0.01 (**A**). Saltiness scores for different solid salts, where different letters indicate significant differences, *p* < 0.05 (**B**).

In order to investigate the dissolution ability of alginate–NaCl and further evaluate its availability in food products, the conductivity changes in alginate–NaCl and NaCl in water were compared. As shown in Figure 6A, the conductivity of the samples showed a rapid increase in the first 5 s, followed by a slower increase. In comparison, the conductivity of alginate–NaCl was significantly higher than that of NaCl within 30 s, which was mainly due to its larger specific surface area increasing its contact area with water. Phan et al. showed that 70 to 95 percent of sodium (or salt) may remain in the food matrix after swallowing, such as potato chips [36]. A quick dissolution of the sodium in the food could contribute to the saltiness perception. Therefore, it is initially speculated that alginate–NaCl had a shorter dissolution time on the taste buds and a higher short-term concentration efficiency, which may provide a stronger saltiness perception at the same additive amount.

### 3.8. Sensory Evaluation of Solid Salts

In order to explore the salt reduction effect of alginate–NaCl, the saltiness of alginate–NaCl was compared with those of NaCl of different amounts by sensory evaluation. As shown in Figure 6B, the saltiness of instant noodles containing 1.5% alginate–NaCl (6.925 ± 0.31 points) was significantly higher than that of instant noodles containing the same amount of NaCl (5 points). Notably, there was no significant difference in saltiness between the instant noodles containing 1.5% alginate–NaCl and those containing 2.0% NaCl (6.85 ± 0.18 points). The above results indicated that compared to the table salt, alginate–NaCl could achieve a certain saltiness in less amounts, and the salt intake could be reduced by about 29% through the application of alginate–NaCl in instant noodles.

Hollow structural design is an effective strategy for sodium reduction. It has been reported that traditional table salt crystals cannot be released thoroughly from the food matrix before swallowing, thus unable to activate taste receptors, while a hollow structure with an amplifying surface area could greatly improve the availability of sodium to taste receptors [2]. Hurst et al. reported that the application of hollow salt in salty snacks, such as potato chips, could significantly reduce the salt intake [37]. The hollow salt prepared in the present study also showed promising potential in the salt reduction. Notably, the application of this technology is economic feasibility due to low cost and easy availability of alginate. Moreover, as a commonly used procedure in industry, spray drying shows its advantages in high efficiency and relative low cost [38]. However, scaling this technology also faces some challenges. For example, hollow particles prepared by spray drying technology are usually in an unfavourable energetic state as a consequence of their large surface area, so they tend to convert to a more energetically favourable state through crystallisation, polymorph transition, crystal growth or particle fusion [39].

## 4. Conclusions

In the present study, hollow salt was fabricated with alginate. It has been revealed that alginate was able to significantly enhance the salty taste of the solutions with the same sodium content and the alginate sample with the highest viscosity (1000–1500 cps) showed the best effect. The mixture of NaCl and HV-alginate was then spray-dried to obtain a solid salt which possessed a particle size of 6.25 ± 2.26 μm and hollow structure without significant alteration in the crystal structure compared to NaCl. Notably, the prepared solid salt demonstrated a higher dissolution rate, and its alginate component could enhance the adhesive retention of sodium ions on the surface of the tongue, which both effectively enhanced the saltiness perception. Furthermore, it was found that alginate–NaCl could reduce salt by up to 29%. The hollow salt obtained in the present study has potential for salt reduction by sprinkling it on solid foods, such as potato chips, barbecue, peanuts, etc., and its wide application may significantly reduce health risks due to excessive salt intake, such as hypertension and coronary heart disease. However, more efforts are still needed to reveal the effect of alginate–NaCl on human health. Moreover, its penetration into the food and its shelf life when applied to the food products also need to be further explored.

## Figures and Tables

**Table 1:** The absorption bands in FTIR spectra and their corresponding functional groups.

No.	Absorption Bands	Corresponding Functional Groups
1	781 cm^−1^	G groups
2	819 cm^−1^	M groups
3	1033 cm^−1^	C-O
4	1418 cm^−1^	carboxyl C=O
5	1619 cm^−1^	carboxyl C=O
6	2928 cm^−1^	C-H
7	around 3400 cm^−1^	lattice vibration, O-H

## Data Availability

The original contributions presented in the study are included in the article/Supplementary Materials; further inquiries can be directed to the corresponding author.

## Supplementary Materials

The following supporting information can be downloaded at https://www.mdpi.com/article/10.3390/foods14010019/s1, Table S1: Specific *p*-values obtained after statistical analysis of the results in Figure 1C.
