# Supplementary material for: Hollow Salt Prepared Through Spray Drying with Alginate Enhances Salinity Perception to Reduce Sodium Intake
Source: Foods. 2024 Dec 25;14(1):19. doi: 10.3390/foods14010019 (PMC11719871; doi:10.3390/foods14010019)
Supplement: Supplementary file 1 [file foods-14-00019-s001.zip › foods-3334963-supplementary.pdf]

# Hollow Salt Prepared Through Spray Drying with Alginate Enhances Salinity Perception to Reduce Sodium Intake

**Qian Jiang, Jiayi Yan, Chen Song, Yunning Yang, Guangyuan Chen, Fanhua Kong, Jingfeng Yang and Shuang Song ***

SKL of Marine Food Processing & Safety Control, National Engineering Research Center of Seafood, Liaoning Key Laboratory of Food Nutrition and Health, School of Food Science and Technology, Dalian Polytechnic University, Dalian 116034, China; rosyqqxi@163.com (Q.J.); 13934546952@163.com (J.Y.); sczoe86@163.com (C.S.); yangyn012@163.com (Y.Y.); noahchen2022@163.com (G.C.); kkong0930@163.com (F.K.); yjfgo@163.com (J.Y.)

* Correspondence: songshuang@dlpu.edu.cn

# Supplementary materials

**Table S1** Specific p-values obtained after statistical analysis of the results in Figure 1C.

| Sample | | P-values |
|---|---|---|
| 0 g/L | LV-alginate-0.25 g/L | 0.026 |
| | MV-alginate-0.25 g/L | 0.002 |
| | HV-alginate-0.25 g/L | 0.000 |
| LV-alginate-0.25 g/L | MV-alginate-0.25 g/L | 0.260 |
| | HV-alginate-0.25 g/L | 0.005 |
| MV-alginate-0.25 g/L | HV-alginate-0.25 g/L | 0.073 |
| 0 g/L | LV-alginate-0.75 g/L | 0.013 |
| | MV-alginate-0.75 g/L | 0.000 |
| | HV-alginate-0.75 g/L | 0.000 |
| LV-alginate-0.75 g/L | MV-alginate-0.75 g/L | 0.028 |
| | HV-alginate-0.75 g/L | 0.000 |
| MV-alginate-0.75 g/L | HV-alginate-0.75 g/L | 0.028 |
| 0 g/L | LV-alginate-1.25 g/L | 0.000 |
| | MV-alginate-1.25 g/L | 0.000 |
| | HV-alginate-1.25 g/L | 0.000 |
| LV-alginate-1.25 g/L | MV-alginate-1.25 g/L | 0.846 |
| | HV-alginate-1.25 g/L | 0.000 |
| MV-alginate-1.25 g/L | HV-alginate-1.25 g/L | 0.000 |
